# Method of delivery of bone marrow stem cells to the articular joint influences their survival during arthroscopy

**DOI:** 10.1186/1471-2164-15-S2-P37

**Published:** 2014-04-02

**Authors:** Reham Al Nono, Gauthaman Kalamegam, Haneen Alsehli, Farid Ahmed, Mohammed Alkaff, Mohammed Abbas, Wael Kafienah, Faten Al Sayes, Adeel Chaudhary, Adel Abuzenadah, Mohammed Al Qahtani, Mamdooh Gari

**Affiliations:** 1 Stem Cell Unit, Centre of Excellence in Genomic Medicine Research, King Abdulaziz University, Jeddah, Kingdom of Saudi Arabia; 2 Department of Orthopaedic Surgery, Faculty of Medicine, King Abdulaziz University, Kingdom of Saudi Arabia; 3 School of Cellular and Molecular Medicine, University of Bristol, UK; 4 Department of Haematology, Faculty of Applied Medical Sciences, King Abdulaziz University, Kingdom of Saudi Arabia; 5 Department of Medical Laboratory Technology, Faculty of Applied Medical Sciences, King Abdulaziz University, Jeddah, Kingdom of Saudi Arabia

## Background

Cartilage poor capacity to regenerate can eventually lead to osteoarthritis. We aim to restore cartilage regeneration by introducing autologus bone marrow MSCs (BMMSCs) into the damaged joint using arthroscopy. The arthroscopic procedure involves variations in temperature either to supraphysiologic or subphysiologic levels following low-flow irrigation or cryotherapy respectively [1,2]. The aim of this study was to assess whether such temperature fluctuations would influence the viability and function of delivered BMMSCs and hence the outcome of the arthroscopic procedure.

## Materials and methods

Primary cultures of human BMMSCs were assessed for their morphology (Phase contrast microscopy), cell proliferation (MTT assay) and surface marker analysis (FACS). Early passage of BMMSCs (P4; 1 x 106 cells/10 mL) were used in two different configurations that reflect their potential method of delivery to the joint: a single cell-suspension (Group A) or a cell-pellet (Group B). The arthroscope with illumination was held in a fixed position such that it was suspended into the medium containing cell-suspension or cell-pellet in 50mL tubes and different samples in both groups were incubated for 10, 20 or 30 minutes. The temperature increased with time from 27.6 ± 0.14 to 37. 2 ± 0.07. The cell-suspension/cell-pellet were then gently mixed and 2 x 10^4^ cells/well (24 well plate) were seeded. Cells were cultured under standard culture conditions (37ºC in 5% atmospheric air) for 72 h and cell morphology and proliferation were assessed.

## Results

**Figure 1:**
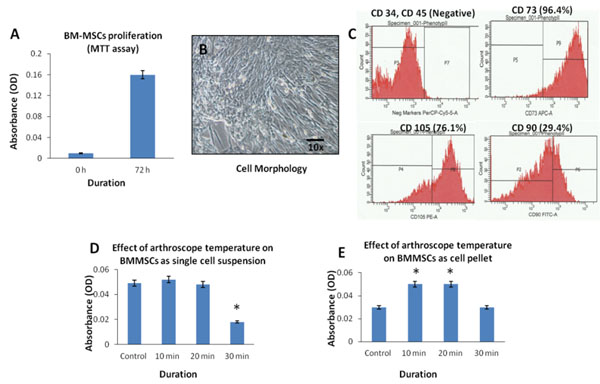
**(A)**. Normal proliferation of BM-MSCs at early passage (P3) by MTT assay; **(B)**. Phase contrast microscopic image of BM-MSCs showing the characteristic short, thin fibroblastic morphology; **(C)**. FACS images showing the positive and negative MSC related surface markers; **(D, E)**. Cell proliferation of BM-MSCs that were exposed to arthroscope either as cell-suspension (D) or as cell-pellet (E). Values are expressed as mean ± SEM (n=3). Asterisk indicates significance (p<0.5) compared to the control.

BMMSCs showed characteristic fibroblastic morphology, proliferation and were positive for BMMSC related surface markers, namely CD73 (96.4%), CD105 (76.1%) and CD90 (29.4%) (Figure 1A-C). They were negative for CD34 and CD45 (Figure-1C). In Group A, assessment of cell proliferation by MTT assay showed decrease by 2.04% and 63.27% at 20 and 30 min, respectively, compared to control following arthroscopic exposure. However, only the decrease observed at 30min was statistically significant (Figure 1D). In contrast, Group B showed statistically significant increases in cell numbers at 10min (33.30%) and 20min (23.33%) compared to the control (Figure 1E).

## Conclusions

Long-term exposure of BMMSCs to the arthroscope as a single cell suspension or in pellets results in decreased cell viability. The pellet configuration seems to confer protection from temperature alterations during short periods of arthroscopic exposure. We conclude that the method of delivery of BMMSC to the joint could be detrimental to their survival and contribution to cartilage repair during arthroscopic procedure.
